# 25 Hz Magnetic Seizure Therapy Is Feasible but Not Optimal for Chinese Patients With Schizophrenia: A Case Series

**DOI:** 10.3389/fpsyt.2018.00224

**Published:** 2018-05-29

**Authors:** Jiangling Jiang, Qingwei Li, Jianhua Sheng, Fuzhong Yang, Xinyi Cao, Tianhong Zhang, Yuping Jia, Jijun Wang, Chunbo Li

**Affiliations:** ^1^Shanghai Key Laboratory of Psychotic Disorders, Shanghai Mental Health Center, Shanghai Jiao Tong University School of Medicine, Shanghai, China; ^2^Brain Science and Technology Research Center, Shanghai Jiao Tong University, Shanghai, China; ^3^Department of Psychiatry, Tongji Hospital of Tongji University, Shanghai, China; ^4^Center for Excellence in Brain Science and Intelligence Technology, Chinese Academy of Science, Shanghai, China; ^5^Bio-X Institutes, Key Laboratory for the Genetics of Developmental and Neuropsychiatric Disorders (Ministry of Education), Shanghai Jiao Tong University, Shanghai, China

**Keywords:** magnetic seizure therapy, schizophrenia, feasibility, acceptability, cognition

## Abstract

Magnetic seizure therapy (MST) is a potential alternative to electroconvulsive therapy (ECT), but there is currently a lack of reports about MST in Chinese patients with schizophrenia. Our objective was to investigate the feasibility and acceptability of add-on MST in the treatment of patients with schizophrenia. Eight patients with schizophrenia were enrolled in a case series study to receive 10 sessions of add-on MST over 4 weeks. The MST was administrated using 25 Hz at 100% output with a titration duration ranging from 4 to 20 s by 4 s. The Positive and Negative Syndrome Scale (PANSS) and the Repeatable Battery for the Assessment of Neuropsychological Status (RBANS) were employed to measure the symptom improvements and the cognitive effects, respectively. Six patients completed at least one-half of the planned sessions. Five showed a reduction in PANSS total score, and three achieved clinical response (≥30% reduction). Three of the participants receiving the RBANS, showed either improvements or no changes in the memory function. Regarding the subjective complaints about MST, two reported dizziness, and only one reported memory loss. Approximately one-fourth of the treatment sessions produced only brief seizures (<15 s). Overall, employing MST to treat Chinese patients with schizophrenia appeared feasible and acceptable. However, further evidence is needed to determine the therapeutic efficacy and effects of MST on the cognitive functions of patients with schizophrenia.

## Introduction

Schizophrenia is generally characterized by marked dysfunctions in cognition, behavior, and emotion (1). Patients with schizophrenia suffer from pronounced function impairment (2), as well as considerable disability (3). While antipsychotics are the mainstay treatment for schizophrenia, ~30% of these patients do not respond fully to pharmacotherapy (4).

Electroconvulsive therapy (ECT) is an important treatment option when the response to pharmacotherapy alone is unsatisfactory or a rapid improvement is desired (5). ECT is widely administered in clinical practice worldwide (6). However, the cognitive side effects, like amnesia and postictal disorientation, are common (7), and the rates of persistent memory loss are between 29 and 55% (8). The substantial impedance of the scalp and skull may contribute to the cognition impairment since it shunts most of the electrical stimuli away, resulting in widespread stimulation of the entire brain (9).

In order to overcome the fundamental shortcomings of the electrical stimuli, the hypothesis of employing magnetic pulses to develop substitutes for ECT was developed in 1994 (10). Due to their ability to pass through the scalp and the skull without resistance, magnetic stimuli can be focused on superficial cortical regions to reduce cognitive side effects (9). Deliberate magnetic seizures were first successfully induced in non-human primates and human beings at the turn of the twenty-first century (11, 12).

The results of animal experiments have indicated that magnetic seizure therapy (MST) induced neither cognitive dysfunction (13, 14) nor pathological changes in the brain (15, 16). Recently, a systematic review confirmed the efficacy and safety of MST on depressive episodes in patients with unipolar depression or bipolar disorder (17). Although the MST parameters varied between the available studies (e.g., pulse frequency, train duration, peak intensity of the magnetic field, and number of treatments), the authors of the review found consistent improvements in the depressive symptoms, the absence of cognitive side effects, and a relatively low remission rate ranging from 30 to 40%. However, significant differences were not found in the direct comparisons of the antidepressant effects between ECT and MST using a randomized controlled design (18, 19). Additionally, studies have also found that MST had superior cognitive outcomes when compared to ECT (20, 21). However, these reports focused only on depression, and there is still a lack of studies concerning MST on patients with schizophrenia.

## Aims of the study

The primary aim of this study was to investigate whether it is feasible and acceptable to treat patients with schizophrenia with add-on MST. We hypothesized that MST could generate antipsychotic effects without significant cognitive or other adverse effects. Our secondary aim was to examine whether MST at 25 Hz could generate adequate seizures in patients with schizophrenia (more than 90% of the treatment sessions achieve a seizure duration of longer than 15 s).

## Methods

### Participants and study design

From February to May of 2016, eight inpatients from Shanghai Mental Health Center in China were recruited into an open-label self-controlled study. The Institutional Review Board of Shanghai Mental Health Center approved the protocol, and the recruitment advertisements were posted in the wards. Those patients who were interested in this study signed the informed consent after being screened according to the study criteria.

Inclusion Criteria: (1) 18–55 years old; (2) Diagnostic and Statistical Manual of Mental Disorders, Fifth Edition (DSM-5) diagnosis of schizophrenia; (3) convulsive therapy clinically indicated, such as severe psychomotor excitement or retardation, suicide attempts, highly aggressive behavior, pharmacotherapy intolerance, and ineffectiveness of antipsychotics (22), as assessed by two attending doctors; (4) the Positive and Negative Syndrome Scale [PANSS; (23)] score ≥ 60; and (5) written informed consent for participating the study and the publication of this case series.

Exclusion Criteria: (1) diagnosis of other mental disorders; (2) severe physical diseases, such as stroke, heart failure, liver failure, neoplasm, and immune deficiency; (3) presenting with a laboratory abnormality that could impact the treatment efficacy or participant's safety; (4) failure to respond to an adequate trial of ECT; (5)pregnant or intend to become pregnant during the study; and (6) other conditions that investigators considered to be inappropriate for participation in this trial (e.g., participating in other clinical trials).

### MST procedure

Generally, the MST setting resembled that of the ECT clinical practice in China (24). In addition to treatment as usual (TAU), the participants were supposed to receive 10 sessions of MST over 4 weeks, with three sessions per week during the first 2 weeks and two sessions per week during the following 2 weeks. The MST was administered under general anesthesia with intravenous etomidate (0.21–0.3 mg/kg) and propofol (1.82–2.44 mg/kg). The muscles were relaxed with intravenous succinylcholine (1 mg/kg), and intravenous atropine (0.5 mg) was employed to reduce airway secretions.

**Figure 1:**
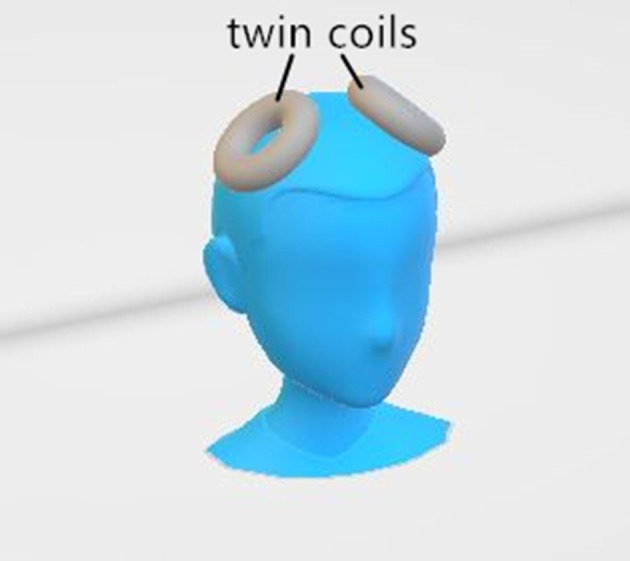
The placement of the twin coils.

The MST was administered with a MagPro X100 (MagVenture A/S, Denmark) at 25 Hz and 100% output. The pulse width was 370 μs, and the peak intensity of the magnetic field was 4.2 Tesla. Given that the seizure threshold is likely to increase as treatment continues (25), a titration method was employed to determine the duration of the magnetic stimulation. It began at 4 sc and was increased by 4 s in each following session up to a maximum of 20 s (i.e., 100 pulses to 500 pulses per session). If the seizure quality was poor (seizure duration of <15 s) in a certain session, the increment of the stimulation duration would be 8 s during the next session. If no seizure was generated, an extra stimulation lasting for 20 s was administrated immediately. The magnetic stimulation was delivered via a twin coil with its midline on the vertex (Figure 1), which was able to induce a stronger and deeper electric field than other MST coil configurations (9).

### Assessments

At the baseline and at the completion of all the treatments, the PANSS and the Repeatable Battery for the Assessment of Neuropsychological Status [RBANS, Form A at the baseline and Form B at the end-point; (26)] were employed to measure the improvements in the psychotic symptoms and cognitive effects, respectively. The response rate was defined as not less than a 30% reduction in the PANSS total score. None of the three patients willing to receive RBANS assessment were able to complete the RBANS subtests involving drawing and writing due to their physical restraints. In addition, memory impairment is one of the most common side effects of ECT; therefore, only the RBANS immediate memory scores and delayed memory scores were presented here. For the patients under 20 years old, their RBANS scores were calculated using the norms for patients of 20–39 years old. In addition to the RBANS, the acceptability was further measured by the adverse events and subjective complains of the participants. The subjective complaints were measured at the end of 10 sessions by asking open questions about the participant's experience during and after the MST. The above-mentioned assessments were conducted by a trained psychiatrist. A Thymatron IV device (Somatics LLC, USA) with left and right frontal leads was utilized to record the electroencephalogram (EEG) during the seizures.

## Results

All eight of the recruited patients were taking atypical antipsychotics, and only one (case 2) was clozapine-resistant. One participant (case 7) dropped out when the family revoked consent because the patient made a suicide attempt the day after the second MST session. One patient (case 8) was excluded because no seizures were generated, even though the maximum stimulation duration was used. Two patients received only eight treatment sessions, one for the recrudescence of persecutory delusion and psychomotor excitement (case 2), and the other for a more rapid relief of psychotic symptoms (case 1). The characteristics of the participants are detailed in Table 1.

**Table 1:** Characteristics of participants.

		**Case 1**	**Case 2**	**Case 3**	**Case 4**	**Case 5**	**Case 6**	**Case 7**	**Case 8**	**Mean (SD)**
Background	Gender	Male	Male	Female	Male	Female	Female	Female	Female	
	Age, years	25	25	17	23	38	21	20	33	25.25 (6.98)
	Education level	U	J	P	P	U	H	H	H	
	Total course of disease, years	5	3	1	3	11	4	2	16	5.63 (5.18)
	Chlorpromazine equivalent, mg	475	666	350	266	367	150	100	150	315.50 (191.16)
MST	Number of treatment	8	8	10	10	10	10	2	1	7.38 (3.74)
	Mean duration of seizures, seconds	22.63	20.88	18.2	17	10.9	15.9	24	0	17.22 (7.01)
PANSS total scores	Pre-MST	99	84	98	96	97	119	93	92	97.25 (10.00)
	Post-MST	50	84	78	64	47	106	NA	NA	71.5 (22.39)
PANSS positive scores	Pre-MST	25	28	26	29	29	32	23	26	66.4 (20.56)
	Post-MST	11	30	11	14	15	21	NA	NA	63.67 (22.03)
RBANS immediate memory	Pre-MST	NA	NA	40	49	85	NA	NA	NA	
	Post-MST	NA	NA	49	49	106	NA	NA	NA	
RBANS delayed memory	Pre-MST	NA	NA	44	44	74	NA	NA	NA	
	Post-MST	NA	NA	44	74	80	NA	NA	NA	

*SD, P, J, H, U, NA, MST, PANSS, RBANS, were the abbreviations of standard deviation, primary school, junior high school, high school, undergraduate, and not available, magnetic seizure therapy, the Positive and Negative Syndrome Scale, and the Repeatable Battery for the Assessment of Neuropsychological Status, respectively*.

**Figure 2:**
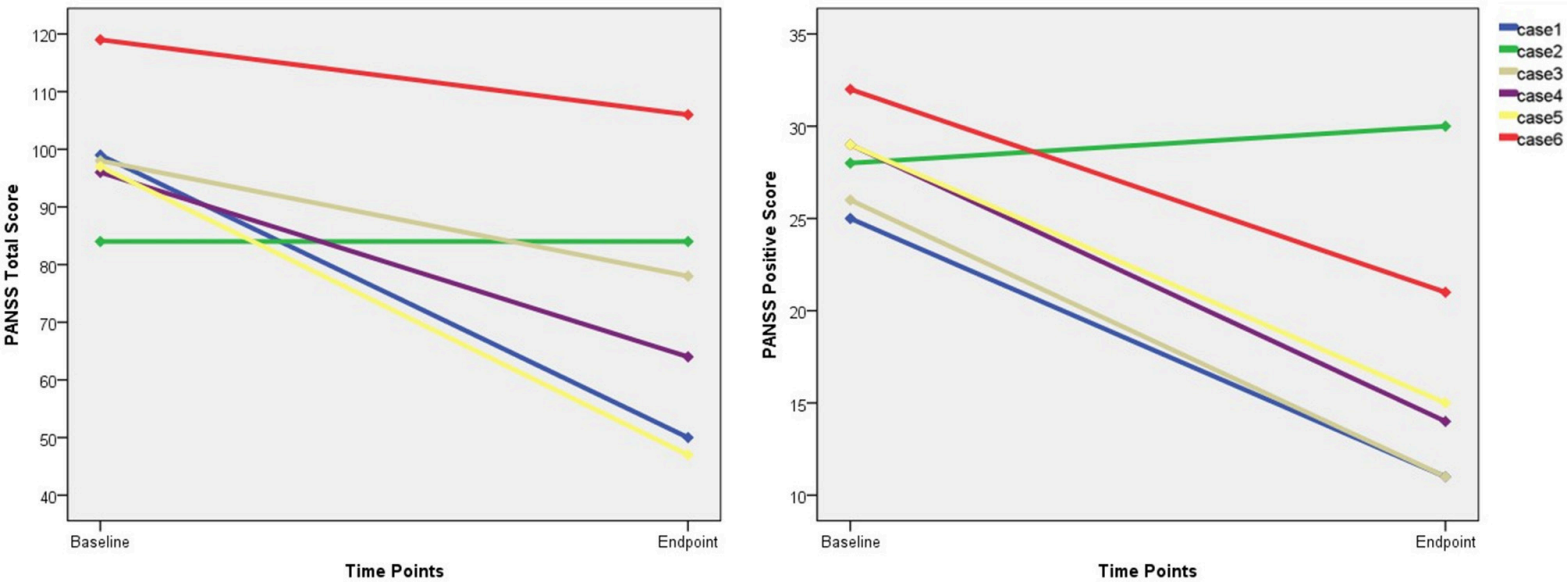
The changes of PANSS total score and positive score. PANSS, Positive and Negative Syndrome Scale.

Five of the six remaining patients who received at least one-half of the planned sessions experienced a reduction in the PANSS total score and positive subscale scores (Figure 2), and three responded to the MST. The PANSS total score and the PANSS positive score (p = .009) showed mean reductions of 27.3 and 11.2, respectively.

**Figure 3:**
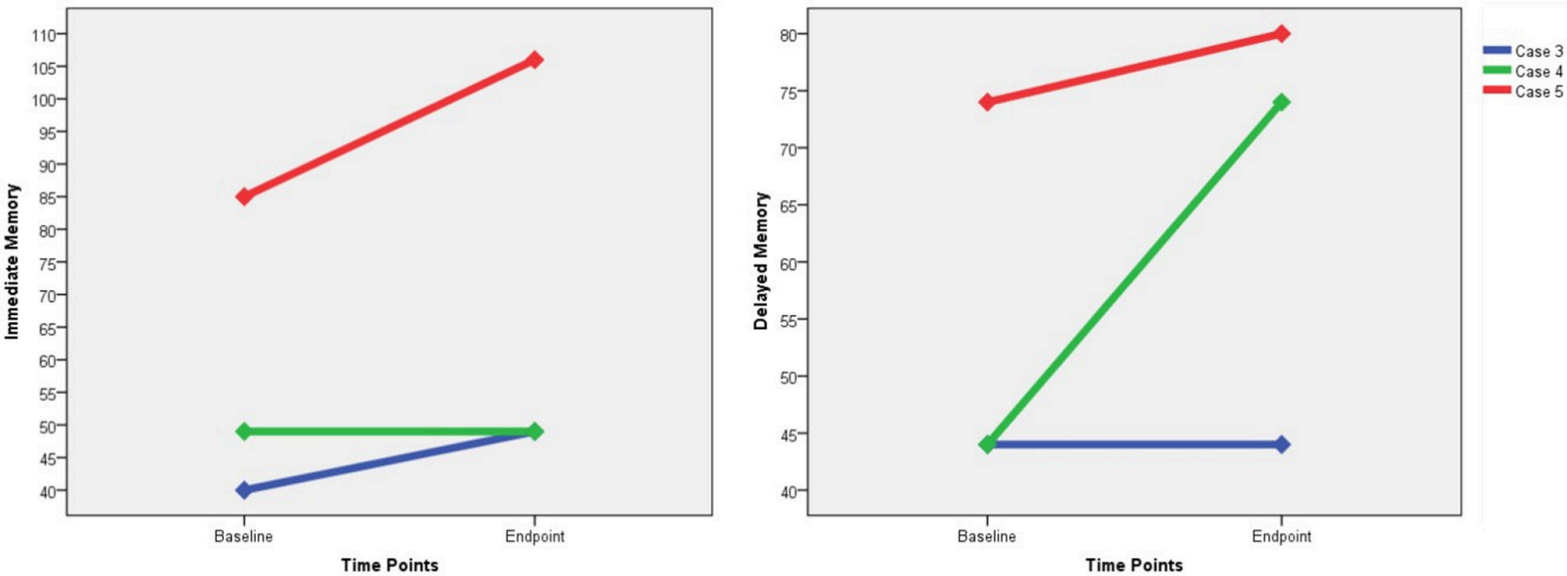
The changes of RBANS immediate memory score and delayed memory score. RBANS, Repeatable Battery for the Assessment of Neuropsychological Status.

Only three participants were capable of receiving the RBANS, and we failed to perform the tests on the other three patients due to their marked auditory hallucinations or functional impairments related to psychotic symptoms. Improvements in the immediate memory were found in two patients (case 3 and case 5), and increases in the delayed memory were found in two patients (case 4 and case 5) (Figure 3). None of these three participants suffered from memory impairment as measured by the RBANS. With regard to the subjective MST experience, two participants reported dizziness (cases 1 and 2), and only one reported subjective memory loss (case 6).

**Figure 4:**
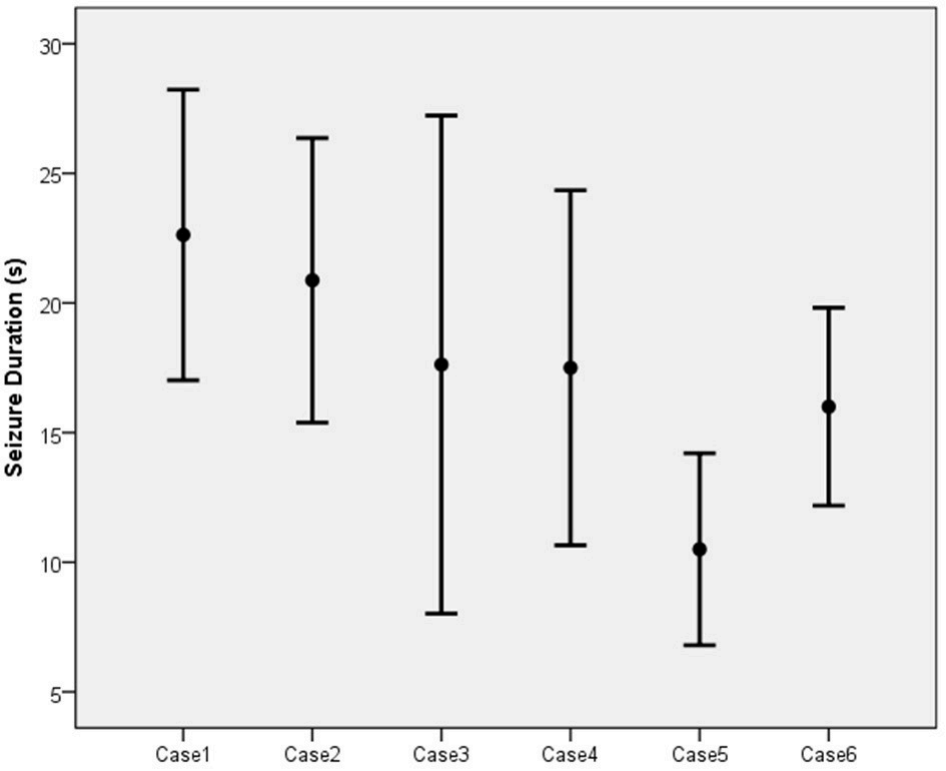
The mean and standard deviation of seizure duration.

The mean duration of the EEG seizure activity in the six patients was 17.29 s with a standard deviation of 6.68 s (Figure 4). Of the 56 sessions completed among the six patients, 13 (23.2%) resulted in inadequate seizures (<15 s). Three patients (cases 3, 4, and 6) each had two inadequate sessions while one patient (case 5) had seven.

## Discussion

To the best of our knowledge, this is the first report of MST conducted on Chinese patients with schizophrenia. The reported response rates (a 50% or more reduction in the score of a certain standardized scale measuring depressive symptoms) of the adjunctive MST on depression ranged from 38.5 to 69.2% (17, 27). When the participants who only received one or two sessions were excluded, our study achieved a similar result that three in six patients with schizophrenia responded to MST. However, when all the participants were included, only 37.5% responded to MST, which was far below the more than 70% response rate to ECT (28, 29). Additionally, we cannot conclude whether MST contributed to the symptom improvement because there were no control groups.

Recently, Tang et al. published the first report of MST used in patients with treatment-resistant schizophrenia (30). They also recruited eight patients in an open-label study. When compared to their study, we applied broader inclusion criteria with the maximum treatment restricted to ten sessions, which are commonly used in the clinical ECT practice among Asian countries (6). Despite the methodological differences, both studies provided preliminary evidence for the antipsychotic efficacy and negligible cognitive side effects. However, the question of whether the efficacy of MST differs according to the combined antipsychotic agents, schizophrenia suntype (e.g., clozapine resistant patients), or the MST parameters requires further investigation.

Kayser et al. reported a 69.2% response rate in depressive patients with mean EEG seizure durations of 19.95 s (27) and the recommended duration was above 15 s (31). However, the mean duration in our study was only 17.29 s with 23.2% of the durations being <15 s. Moreover, there was a “missed-shot” (no seizures generated) in the patients who completed all ten treatment sessions. The poor seizure quality was likely to have contributed to the poor response rate (32).

When compared with 50 and 100 Hz, it is reported that 25 Hz of MST is superior in the seizure quality with an equal response rate for patients with treatment-resistant depression (33). Nevertheless, the seizure quality was generally poor in our trial with a 25 Hz stimulation on patients with schizophrenia. There was a patient in whom even the magnetic pulses with the maximum stimulation duration of our protocol were unable to induce any seizures. It must be noted that this participant was the only one with previous ECT treatments, which may have led to the rise of the seizure threshold (25). Thus, we will try higher frequencies in future trials to see if this can improve the seizure quality and therefore the response rate. Additionally, combined antipsychotics and other medications can also affect the seizure characteristics of ECT (34). All of our participants were taking atypical antipsychotics, but we still cannot rule out these medications contributing differently to the brief seizure duration. Therefore, the effects of combined medications on MST need to be considered in future studies.

Like the previous studies (20, 21), no evidence of cognitive impairment was found in the patients treated with MST. It was impossible to determine the mixed results of the cognitive performance with such a small sample size, but the memory scores were unaffected or even improved. Only one of the patients complained of memory problems, and this participant obtained the worst scores in the post-treatment PANSS. The performances in autobiographical recall (35) and delayed verbal recall (36) have been shown to be selectively impaired after ECT. However, we found no delayed recall reduction following the MST while Tang et al. (30) revealed cognitive side effects in the autobiographical recall without a deterioration in the working memory, executive function, or other cognitive domains that are commonly impaired among patients with schizophrenia. It is noted that we fixed the frequency of the magnetic stimulus at 25 Hz and the number of treatment sessions at ten, while Tang et al. employed a flexible frequency from 25 to 100 Hz with a maximum treatment of 24 sessions. Based on these very limited cues, it may be useful to improve the seizure quality and treatment response by employing higher magnetic pulses dosages and frequencies. Nevertheless, the effects of the frequency, number of treatment, and other parameters on the cognitive functioning require further investigation.

One patient attempted suicide after receiving two MST sessions. This patient was a 20-year-old undergraduate student, and she was hospitalized for persecutory and hypochondriac delusions. She already exhibited suicidal ideation and several self-injurious behaviors before admission. Since a previous study indicated that MST could significantly reduce the suicidal ideation in depressive patients (37), the suicide attempt was unlikely to be a side effect of the MST. No other adverse events were identified, and the most common side effects were dizziness. Therefore, the preliminary evidence suggested that the MST was acceptable in patients with schizophrenia. However, more studies are needed to determine the relationship between MST and suicide in patients with schizophrenia, and to investigate the safety profile of MST.

This preliminary research was limited by the small sample size and the lack of a controlled group, which caused difficulties in interpreting the psychotic symptom improvements, analyzing the cognitive function test results, and investigating the potential factors for the poor seizure quality. In future studies, we plan to determine testify whether higher dosages (frequencies) can produce more adequate seizures. A randomized double-blind ECT-controlled design with an optimal sample size will also be employed to further investigate its efficacy and safety. Additionally, the peripheral cytokines, EEG measured event-related potential, and structural and functional magnetic resonance images will help us to understand the physiological effects of MST on the brains.

## Author contributions

QL, JS, FY, XC, and TZ collected the data. JJ analyzed the data and drafted the manuscript. YJ, JW and CL contributed to the study design and revised the manuscript critically. All the authors approved the final version of the manuscript.

## Acknowledgments

The present study was supported by the grants of Shanghai Hospital Development Center (SHDC12014111), the Science and Technology Commission of Shanghai Municipality (13dz2260500, 14411961400, and 17411969900), Shanghai Municipal Commission of Health and Family Planning (201740042), and the SHSMU-ION Research Centre for Brain Disorders.

### Conflict of interest statement

The authors declare that the research was conducted in the absence of any commercial or financial relationships that could be construed as a potential conflict of interest.
